# Zinc is an Essential Element for Male Fertility: A Review of Zn Roles in Men’s Health, Germination, Sperm Quality, and Fertilization

**Published:** 2018

**Authors:** Ali Fallah, Azadeh Mohammad-Hasani, Abasalt Hosseinzadeh Colagar

**Affiliations:** -Department of Molecular and Cell Biology, Faculty of Basic Sciences, University of Mazandaran, Babolsar, Iran

**Keywords:** Germination, Male fertility, Microelement, ROS, Sperm parameters, Zinc

## Abstract

Zinc (Zn) is the second most abundant trace element in human, which can’t be stored in the body, thus regular dietary intake is required. This review explained the physiological and pathogenesis roles of zinc in men’s health and its potentials in germination, quality of sperm, and fertilization. Our investigation showed that Zn contained many unique properties in human, especially males. The antioxidant quality is one of them. Also, the increased reactive oxygen species levels in the seminal plasma of men who are both infertile and smokers influence the Zn content of seminal plasma in a way that physiology of spermatozoa can be affected as well. Moreover, Zn acts as a toxic repercussionagainst heavy metals and cigarette inflammatory agents. Zinc as a hormone balancer helps hormones such as testosterone, prostate and sexual healthand functions as an antibacterial agent in men’s urea system. It plays a role in epithelial integrity, showing that Zn is essential for maintaining the lining of the reproductive organs and may have a regulative role in the progress of capacitation and acrosome reaction. In contrast, Zn deficiency impedes spermatogenesis and is a reason for sperm abnormalities and has a negative effect on serum testosterone concentration. Based on these findings, Zn microelement is very essential for male fertility. It could be considered as a nutrient marker with many potentials in prevention, diagnosis, and treatment of male infertility.

## Introduction

Sexual accessory glands secrete in the seminal plasma containing some elements that protect spermatozoa at the time of ejaculation (1). It consists of proteins, such as many enzymes (Acid phosphatase, alanine transaminase, alkaline phosphatase, aspartate transaminase), lipids, macroelements [Sodium (Na+), Potassium (K+), Calcium (Ca2+), Magnesium (Mg2+), Phosphate (P) and Chloride (Cl)] and microelements [Copper (Cu), Iron (Fe) and Zinc (Zn)] (2). Zn is a plentiful microelement in the body and it is found in nuts, legumes (3), seafood (*e.g*. Oysters), fortified cereals, cremini mushrooms, low-fat yogurt (4) and animal proteins, such as meat, fish and milk. Some reports showed that consumption of these natural foods can enhance germinal cell proliferation (5), and poor Zn nutrition may be an important risk factor for the low quality of sperm and idiopathic male infertility (6). Dietary Zn deficiency (less than 5 ppm) impairs reproduction in males and females. On the other hand, World Health Organization (WHO) estimates that one-third of world population has a Zn shortage (7). Zn is necessary for body’s proper physiological functions like, normal growth, reproduction, DNA synthesis, cell division and gene expression, photochemical processes of vision, wound healing, ossification, and augmenting the immune system of the body (8–10).

Zn concentration is high in the seminal fluid and has a multifaceted role in the sperm’s functional properties. It has been suggested that Zn acts as an important anti-inflammatory factor and that it is involved in the sperm’s oxidative metabolism. Zn has many important functions in the spermatozoa physiology, including effects on lipid flexibility and sperm membrane stabilization (11). It also has a regulated role in capacitation and the acrosome reaction of sperm and is essential for conception and embryonic implantation (12).

In human spermatozoa and probably in the rest of the mammalian species, the Zn finger motif Cys2/His2 of P2 protamine has an important role in prevention of transcription through sperm chromatin stabilization and in the inhibition of oxidative damage. Proteins containing Zn and selenium in mammalian spermatozoa are involved in modulating the amount of reactive oxygen species (ROS); therefore, it is interesting to investigate whether balanced amounts of Zn and other oligoelements that are added to the semen diluent would moderate the deleterious effects of ROS on chromatin structure (13). Contreras et al. demonstrate that a concentration of 200 ppm from Zn supplementation had an adverse effect on boar sperm DNA quality and it could be related to the capability of spermatozoa to cumulate Zn during spermatogenesis. They also showed between percentage of sperm DNA fragmentation (SDF) and Zn concentration in spermatozoa a positive relation (13–16).

In our previous study, it was demonstrated that an important risk factor for low quality of sperm and idiopathic male infertility is poor Zn nutrition (6). But in this review, the focus was on the various roles of Zn in male health, reproductive system, quality and function of sperm, as well as the effects of epigenetic factors on Zn and finally the molecular and cellular mechanisms of Zn.

### 1. Roles of Zn in health

The human body contains 2–3 grams (2000–3000 milligrams) of Zn and nearly 90% is found in muscles and bones. The nearby organs include prostate, liver, the gastrointestinal tract; kidney, skin, lung, adrenals, brain, heart, eyes and pancreas contain estimable concentrations of Zn. Blood tests for Zn deficiency are inaccurate because majority of Zn is cumulative inside cells and is not free in the blood. There are several reasons that Zn is important to men’s health. Assisting immune function, patronage of healthy cell growth, having a role in preserving prostate health, sexual health and testosterone hormone levels are typical examples. It has been demonstrated that Zn plays a significant role in reproductive functions (14, 17). Hosseinzadeh Colager et al. reported Zn levels in fertile groups for whom the seminal plasma was significantly higher than the level in infertile groups. The level of Zn in fertile men was 14.08± 2.01 and in the infertile group was 10.32±2.98 (*mg*/100 *ml*) (6).

There are many reports that showed reduction or increase in consumption of Zn which let to overindulge vis-a-vis deficiency damages in many of the human organs (Table 1). For example, Zn deficiency is correlated with reduction in testis volume, a decrease in testicular weight, hypogonadism, gonadal dysfunction, inadequate development of secondary sex specifications in human, shrinkage of seminiferous tubules, the failure of spermatogenesis, male gonad growth and hypogonadism (8). And so, there are many reports for Zn-physiological roles in human (Table 2), including its role in gonads- and some enzyme-functions, treatments of some diseases, and better function of apoptosis and immune systems. Some of these Zn-physiological roles are indicated below.

**Table 1.** Comparisons of the effects of zinc overindulge vis-a-vis deficiency^*^

**Deficiency <9 *μM***	**Excess >17 *μM***
Brain	Brain
Decreased nerve conduction	Lethargy
Neuropsychiatric disorders	Focal neuronal deficits
Neurosensory disorders	Respiratory Tract
Mental lethargy	Respiratory disorder after inhalation of zinc smoke
Thymus	Metal fume fever
Thymic atrophy	Gastrointestinal tract
Reduced activity of the thymus gland	Nausea/vomiting
Reduction in the activity of thymulin	Epigastric pain
Pituitary-, pineal- and hypothalamus gland	Diarrhea
Impaired synthesis/secretion of FSH and LH	Prostate
Decreased binding of oxytocin to its receptor	Elevated risk of prostate cancer
Abnormal leptin levels inhibiting the release of NPY	Associated Disorders
Thyroid gland	Alzheimer’s disease
Reduced conversion of thyroxine to triiodothyronine	Autism spectrum disorders
Skin	Epilepsy
Skin lesions	ADHD
Decreased wound healing	Mood disorders
Acrodermatitis	Schizophrenia
Reproductive system	ALS
Infertility	Friedreich’s ataxia
Retarded genital development	Multiple sclerosis
Hypogonadism	Diabetes
Changed sex steroid hormone receptor levels	Sickle cell anemia
Modification of testosterone levels (excessive conversion of testosterone into estradiol)	Acrodermatitis enteropathica

* Concluded from previous studies (14, 16, 18)

**Table 2.** Physiological roles of zinc in human^*^

Dermal exposure	Gonads
Applying zinc preparations on skin protects against skin burns and wind burns, have antidandruff properties and is an important ingredient of shampoos	Help the development of gonads and normal growth, act as key factor in prostate gland function, increase testosterone production and finally improve spermatogenesis
Treatment	Enzyme function
Helps in the treatment of bacterial infection like shigellosis, also improves the helping of viral infections in zinc deficient individuals	Helps in the normal function of large number of enzymes and acts as the core ion of their reaction center
Brain	Immune system
Helps improving cognitive abilities, neurotransmission and synapse formation	Zinc helps improving immune activity, while deficiency leads to delayed response to both T cell dependent and T cell independent antigen. Further, its deficiency decreases ratio of T4+ and T8+, IL-2 and NK cell lytic activity
Apoptosis	
Helps preventing apoptosis	

* Concluded from previous studies (14–16)

#### 1.1. The role of Zn in immunity:

Zn contains many roles in the immune systems (Table 3). Cells’ innate immunity, neutrophils, macrophages and natural killer cells needed Zn for ordinary growth and its deficiency in function has negatively affected the development and function of T and B cells, phagocytosis, intracellular killing, and cytokine production. Zn has a role in almost all aspects of inherent immunity and compatibility, so Zn deficiency may influence weakening of immune system and reproductive system. Zn is effective in various mechanisms, including DNA replication, RNA transcription, proliferation, and differentiation and activation of immune cells in many organs of the body (9, 10, 19).

**Table 3.** The main effects of zinc deficiency on the immune system^*^

PMN-Cells	B- Cells
Reduced number of PMN cells	Reduced number of B- cells
Reduced chemotaxis of PMN cells	Decrease antibody production
Monocytes	Pre-T- Cells
Reduced number of monocytes	Impaired maturation of Pre-T- cells
NK-Cells	Reduced number of T- cells
Decreased lytic activity of NK cells	Macrophages
T-Cells	Diminish activation of macrophages by T helper cells
Reduced cytotoxic action of T-cells	

* Concluded from previous studies (14–16, 20, 21)

The innate as well as the specific parts of the immune system are influenced by Zn. The effects of Zn are multifaceted. Zn can induce adhesion of myelomonocytic cells to the endothelium, while Zn chelation diminishes cell recruitment. Thus, Zn is essential, even in the earliest stages of an immune response. At the molecular level, Zn is required for the interaction between the p58 killer cell inhibitory receptor on NK cells and major histocompatibility complex (MHC) class I molecules, mainly human leukocyte antigen C, on target cells, resulting in the inhibition of the killing activity (8). Interestingly, Zn is needed to maintain the normal function of natural killer cells, and Zn deficiency may result in non-specific killing activity and functional loss. In addition to effects of upstream signaling molecules, Zn influences gene expression by structural stabilization and functional regulation of various immunological-relevant transcription factors as summarized earlier (20). Zn corrects these adverse effects of cadmium on the immune system and biochemical processes as a component of the antioxidant superoxide dismutase and protects the testicular endothelium and sperm cell membranes (21).

### 2. Roles of Zn in male reproductive system

#### 2.1. Zn in testis:

Zn exists at high levels in the testis of vertebrates which are comparable to liver and kidney. Moreover, there are certain reports that manifest Zn can reduce testis injury by stresses such as heavy metals, fluoride, and heat (22). Zn is assembled in the testis during early spermatogenesis and may play a main role in the adjustment of the spermatogonial reproduction and in the meiosis of germ cells (23). Mostly, Zn assembles in germ cells and its concentration in testis increments during spermatogenesis. That’s why Zn is not detectable in either interstitial tissue or sterol cells and that a Zn deficiency impedes spermatogenesis which is the reason for sperm abnormalities (5).

#### 2.2. Zn and male hormonal statue:

Some researchers have witnessed that Zn is needed for the normal functioning of the hypothalamus-pituitary-gonadal axis. Because of the important role of Zn in male reproductive potential, it is paramount to recognize that andrological variations which are most sensitive to Zn depletion. An evaluation of those variables in clinical cases of possible Zn deficiency would accelerate treatment. Zn influences male fertility in several different ways. Low Zn levels have a negative effect on serum testosterone concentration (24).

Thyroid hormones have several important roles in the body including metabolism, development and even body temperature. Zn helps the body maintain proper thyroid function by producing hormones in the brain called thyroid-releasing hormones. Whenever men are low in Zn, they may fail to produce enough of these hormones. That can also affect testosterone levels (4). A clinical study indicated that adult males who denied Zn showed a disorder of testosterone synthesis in the Leydig cell, since Zn has a main role in the 5α reductase enzyme that is necessary for the transformation of testosterone into a biologically active form, 5α dihydro testosterone (25). Insomuch that Zn influences a man’s fertility, sex drive and long-term sexual health are influenced. An investigation of Zn’s influence on testosterone hormone levels showed that catching Zn complementary enhanced testosterone output (4). Seminal Zn is considered as an indicator of prostatic function; however, the function of Zn in seminal plasma and semen is unknown. Most Zn secreted from the prostate in humans seems to target the proteins secreted from seminal vesicle. Other research shows that the Zn and albumin secreted from the prostate constitute a complex that covers the sperm and thereby protects the cells (24).

Zn is situated primarily in the Leydig cells, the late type B spermatogonia, and the spermatids. And also is vital for the production and secretion of testosterone from the Leydig cells. Zn in human semen seems to play an important role in the physiology of spermatozoa. On the other hand, there is remarkable evidence that Zn deficiency causes primary testicular failure, lessens function of the luteinizing hormone receptor, lessens steroid synthesis and Leydig cell damage due to oxidative stress (26).

#### 2.3. Zn and prostate:

The prostate compared to other tissues and body fluids has high concentration of Zn. Actually, Zn is a marker of prostatic function. Its other roles are regulation of the functions of spermatozoa, acting as a co-factor for most enzymatic reactions, and helping in preservation of sperm motility (17). Zn as a downward molecular weight complex with citrate or bound to glycoproteins of the sperm vesicles is discharged from the prostate demonstrating that biologic Zn therapy has a positive effect on sperm motility and the use of biologic Zn supplementation was an efficient method for the treatment of infertile males with chronic prostatitis (27).

#### 2.4. Antibacterial activity:

Zn oxide nanoparticles have bactericidal effects on both gram-positive and gram-negative bacteria and are also effective against spores which are resistant to high temperature and high pressure (28, 29). Prostatic Zn may have antibacterial activity because trichomonas vaginalis is easily killed at concentrations of Zn that occur in the prostatic fluid of healthy men (24). Furthermore, Zn has antibacterial activity and antilipid peroxidation properties that maintain sperm membrane stability and protect the testis against the degenerative changes (12).

#### 2.5. Roles of Zn in quality and function of sperm:

Evidence suggests that seminal Zn has an important role in the physiologic functions of the sperm and that its reduced levels result in low seminal quality and subsequent chances of fertilization (6).

##### 2.5.1 Sperm functions:

Zn is known to be essential for sexual maturity and onset of estrus. Zn plays a role in epithelial integrity, showing that Zn is essential for maintaining the lining of the reproductive organs (6). Furthermore, it has an important role in stabilizing the cell membrane and nuclear chromatin of spermatozoa in seminal plasma. It may have a regulative role in the progress of capacitation and acrosome reaction. The Zn concentration in human seminal plasma is higher than in other tissues (30). Zn has been shown to be vital for spermatogenesis. It plays a significant role in testis development and sperm physiologic functions (6). Zn contains a variety of roles in the spermatogenesis phases (Table 4). For example, in the initiation of spermatogenesis, Zn is important in participation of ribonuclease activities that is highly active during mitosis of spermatogonia and meiosis of spermatocytes. During spermatogenesis, spermatids obtain tail and motility with a developed mid-piece which connects the head compartment with the tail. This physical maturation process occurs in seminiferous tubule. Sperm maturation and storage happens in the epididymis. At the end of spermatogenesis, Zn is highly concentrated in the tail of mature spermatozoa and involved in sperm motility (30).

**Table 4.** Roles of Zn during various phases of spermatogenesis

**Initiation**	**During**	**The end**
Participation of ribonuclease activity	Involves in spermatozoa maturation Maintains germinal epithelium and seminiferous tubule	Enhances sperm motility

Though the dynamics of Zn during sperm expansion is far to be ambiguous but, the significant role of Zn in normal spermatogenesis and sperm suitability is known. Foresta et al. explained the expression of Zn transferor at testicular, epididymis and ejaculate levels and realized the role of Zn content at different steps of sperm life in communication to sperm role (31). Optimal concentrations of Zn in the seminal plasma have also been related to an increase in sperm concentration of the ejaculate (11), high motility, viability (32) and enhancing antioxidant activity (33). A low concentration of Zn has been indicated to disturb the fatty acid composition of the testis and interfere with normal endocrine regulation of the testis (34). Zn is associated with catabolism of lipid that is done in sperm middle piece and provides energy for motion of spermatozoa. Zn effects oxygen consumption of the spermatozoa in seminal plasma and also influences head-tail attachment or detachment and nuclear chromatin condensation/decondensation. Testicular Zn is crucial for normal spermatogenesis and for sperm physiology; it maintains genomic integrity in the sperm and fixes attachment of sperm head to tail (32).

The effects of Zn ions on the expression of germ cell genes from BMMSCs were investigated by Ghasemzadeh-Hasankolai et al. Zn ions can increase male fertility by regulation of the expression of testis GC-specific genes during the differentiation process and spermatogenesis (35, 36). Also Zn therapy reduced asthenozoospermia (37).

##### 2.5.2. Sperm quality:

Supra-nutritional doses of Zn, Co, and Se were used to enhance the production of motile sperm with intact membranes (38). Several studies have demonstrated that oral Zn supplementation improves sperm motility in subfertile men with idiopathic asthenozoospermia and/or oligozoospermia (24). The negative correlation between seminal plasma Zn and sperm viability is a good sign of the importance of Zn in spermatogenesis (39). This may be clarified by the necessary role of Zn in protein metabolism and nucleic acid synthesis. Alteration of seminal plasma Zn changes count, motility, viability, pH and viscosity. Seminal plasma Zn-T (Zn per ejaculate) is the better marker for assessing the relationship between Zn and semen quality (40). Low seminal Zn levels have been related with decreased fertility potential. Furthermore, it was indicated that oligospermic men with sperm counts <20 million cells per milliliter had slightly lower seminal plasma Zn concentrations compared with normozoospermic men. However, other authors reported that normozoospermic and oligoasthenozoospermic patients had similar seminal plasma Zn concentrations (41). Akinloye et al. showed that a low level of Zn in cells is a contributing factor to spermatogenesis reduction and low cellular testosterone in infertile Nigerian males (39). There is extensive evidence that human seminal Zn has an important role in the physiologic sperm functions and Zn reduced levels led to low quality of sperm and reduced fertilization chances. Some authors have reported that high Zn concentration are correlated with enhanced sperm parameters, including sperm count, motility, and normal morphology (11, 12, 42), whereas in Danscher et al.’s study, a high concentration of Zn is correlated with poor sperm motility (43). Zhao and Xiong observed a lower content of Zn in the seminal plasma of infertile subjects and a positive relationship with poor sperm production and poor sperm motility (44). Many studies could not find a significant dependence between total Zn in seminal plasma and sperm quality (17, 45). Hosseinzadeh Colgar et al. showed that, seminal Zn had a significant positive correlation with sperm count and normal morphology in fertile and infertile men (6). Chia et al. found seminal plasma Zn concentrations are directly associated with sperm density, possibly contributing a positive effect on spermatogenesis (11) Henkel et al. have shown the effects of Zn on sperm motility, confirming the mineral’s role in flagella function (46). Serum Zn levels had significant positive correlations with sperm count and sperm motility and seminal plasma Zn concentrations were closely associated with sperm density and motility; these observations were consistent and also no significant correlation of sperm morphology with seminal plasma Zn concentration was found (12, 47). Fuse et al. observed a positive correlation between Zn levels and sperm motility. Hussain et al. reported a significant low level of seminal plasma Zn levels in oligozoospermic and azoospermic males. These findings are consistent with the results of Hasan et al.’s report and against studies of Wong et al.; however, Hussain et al. found a positive correlation between the sperm count and seminal plasma Zn concentration. This element has been shown to be highly significant for conception, successful implantation and pregnancy outcome (25, 48). Henkel et al. have shown the effects of Zn on sperm motility, emphasizing the mineral’s role in flagella function. Zn therapy improves sperm quality with increases in sperm density, progressive motility and improved conception and pregnancy outcome. Zn plays an important role in membrane-stabilizing and antioxidant activity and maintains sperm viability by inhibiting DNases (37, 46).

Several studies have reported that seminal Zn concentration is associated with sperm count, (42, 49) and that poor Zn nutrition is a significant risk factor for low quality of sperm and idiopathic male infertility (6). On the other hand, other studies have observed no significant association between Zn and semen quality parameters (12, 45). Badade et al. believe that, there is the positive correlation between Zn with sperm count and sperm motility. It indicates an important role of Zn in spermatogenesis. Therefore, these parameters could be beneficial for characterizing sperm fertilization potential in diagnosis, anticipation and treatment of male infertility particularly in idiopathic male infertility. This beneficial effect of Zn may be due to Zn-induced reduction in the plasma oxidative stress intensity and modulations of the immune response (50, 51).

Zn sulfate treatment in serum and seminal plasma level of anti-sperm antibody (ASA) have result in a significant enhancement of the seminal fluid parameters including sperm concentration, sperm motility, vitality and normal sperm morphology (52). Khan et al. have demonstrated that, a decrease in seminal Zn concentration influences sperm count, while increased level of seminal plasma Zn decreases sperm motility; this suggests that seminal plasma Zn levels should be very carefully measured in patients who have low sperm count but normal sperm motility, since adequate seminal Zn is required for normal sperm function (3). Although few older studies rejected effects of Zn on sperm quality, many recent studies accept the relation between Zn and motility, morphology or sperm count. Perhaps one of the reasons for this correlation is the existence of Zn in the nucleus and tail of early sperm for chromatin condensation and motility. Some of the main functions and effects of zinc deficiency in the male reproductive system are shown in table 5.

**Table 5.** The main functions and effects of zinc deficiency in the male reproductive system^*^

**Function**	**Deficiency**	**Conclusion**
Acrosin activity Acrosome reaction Testosterone synthesis Testicular development Testicular steroidogenesis Sperm chromatin stabilization Nuclear chromatin condensation Oxygen consumption of spermatozoa Manufacture 5α-DHT^**^ from testosterone	Hypogonadism Gonadal dysfunction Shrinkage of seminiferous Failure in spermatogenesis Decrease Testicular weight Arrest of testis development Retarded genital development	Enhancing sperm mobility through ATP system and phospholipid Reduction in the incidence of anti-sperm antibodies Improvement in sperm quality and sperm motility Fertilization capacity

* Concluded from previous studies (14, 15, 30);

** 5 α-di hydro testosterone

### 3. Epigenetic and Zn levels

Epigenetics eventuate in the transmission of information from a native cell to a girl cell without the precise coding of information in the DNA sequence. In fact, this process can be done by removing or adding specific molecules from the DNA (53).

Actually DNA methylation makes genes turn on or off and determines which proteins are transcribed. Also, this change is an epigenetic mechanism. It happens when methyl groups are combined into cytosine molecules by DNA methyl-transferases (DNMTs), creating 5-methylcytosine. This process provides silencing of genes. Epigenetic mechanisms are associated with gene expression of Zn transporters and Zn binding proteins (54, 55).

#### 3.1. Effects of oxidative stress on Zn levels of seminal plasma:

Many studies have shown that Zn has antioxidative activities and has a main role in scavenging ROS. ROS (*e.g*.: superoxide [O_2_^−^] anion, hydrogen peroxide [H_2_O_2_], peroxyl [ROO^−^] radical and the very reactive hydroxyl [OH^−^] radical) are unstable compounds with a short half-life that can adversely influence certain cellular functions. High ROS levels affected sperm function by oxidation of lipids, proteins, and DNA (56–58).

**Figure 1.**
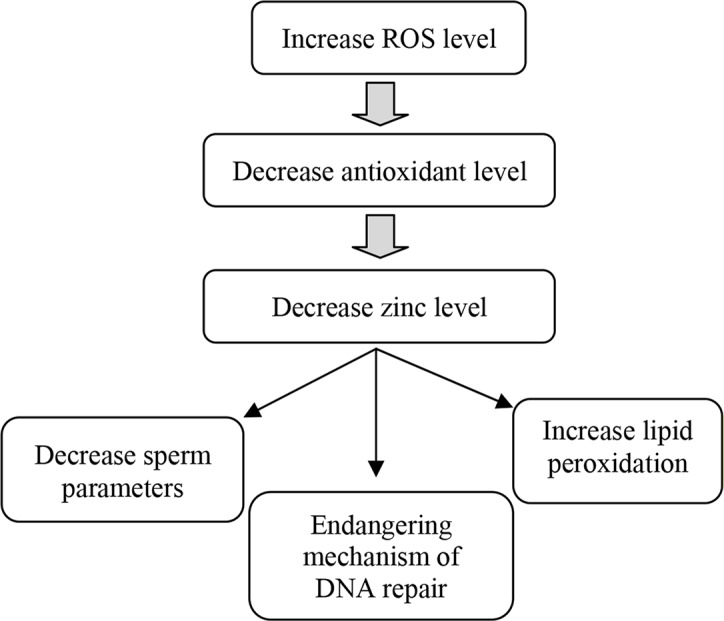
Effects of increasing ROS level in seminal plasma

Studies show that Zn has antioxidative activity. So that oxidative damage due to ROS can be an increase in Zn deficiency. Zn is a necessary component of Cu/Zn superoxide dismutase, which has antioxidative activity for sperm function (56). A small amount of ROS is essential for sperm to obtain fertilizing capabilities, but high seminal ROS levels may reduce the effective concentration of seminal Zn (59). Increased ROS levels in the seminal plasma of infertile men may decrease Zn concentration, increasing detrimental effects of ROS to sperm cells that are correlated with abnormal sperm parameters (Figure 1). Several studies suggested the hypothesis that decreased Zn concentration can lead to an increase in oxidation of DNA, proteins and lipids which underlined Zn main role in prevention of oxidative damage. Zn has been shown to have antioxidant properties to maintain sperm viability by inhibiting DNase activity. Zn is present at high concentrations in the seminal fluid and there is evidence that Zn appears to be a potent scavenger of excessive generation of superoxide anions produced by abnormal spermatozoa and/or leukocytes in human semen after ejaculation. The abnormal spermatozoa would be a source of superoxide anions that bind with Zn present in the seminal plasma and thus decrease the Zn levels. Therefore, it may not be helpful to use infertile patients only to study the correlation between semen parameters and Zn concentrations in seminal plasma and blood (11, 56, 59).

#### 3.2. Effects of cigarette smoking on Zn levels of seminal plasma:

Cigarette smoking has been shown to influence sperm quality, including sperm concentration and spermatozoal morphology, especially sperm head piece. A number of studies have shown that cigarette smoking has a harmful effect on sperm quality, most significantly sperm concentration, motility and morphology; however, some studies have found no relationship between smoking and sperm parameters. Such conflicting results could be due to the fact that the studies were conducted on different populations (60) Kumosani et al. showed that cigarette smoking influences both Ca^2+^-ATPase activity and the motility of spermatozoa, by increased seminal cadmium and Zn concentrations reduction. Therefore, it has been indicated that cigarette smoking can increase oxidative damage via decreasing seminal plasma Zn concentration and other antioxidants, thereby affecting sperm parameters (61). Liu et al. illustrated a negative relationship between cigarette smoking and seminal plasma Zn concentration. Heavy smoking was associated with low sperm count, low motility, poor morphology and increased seminal cadmium levels so that Zn therapy improved sperm quality. (62). Mankad et al. have shown that Zn has a possible role in sperm membrane stabilization (42). Kiziler et al. reported that there is a relationship between oxidative damage resulting from smoking and antilipid peroxidation of seminal plasma Zn (63). Liu et al. found that cigarette smoking adversely affected sperm count, motility and morphology and this observation was supported by other studies (62). Sperm concentration, motility and morphology in smokers with abnormal Zn levels were significantly lower than in all non-smokers, regardless of Zn levels, supporting a previously reported relationship between seminal Zn levels and sperm parameters (34, 42, 61).

Hosseinzadeh Colagar et al. compared the Zn levels in the seminal plasma of fertile and infertile men, smokers and nonsmokers. Smokers (fertile or infertile) had lower levels of Zn in their seminal plasma than nonsmokers. This difference was not significant, but they also showed that smokers are susceptible to Zn deficiency in their seminal fluid. The depletion mechanism of Zn in the seminal plasma of smokers and infertile patients has not been fully understood (6). Recently, studies have suggested that cigarette smoking increases seminal ROS due to several occurrences: (i) The high level of ROS in cigarette smoking and (ii) smoking metabolites may induce an inflammatory reaction in the male genital tract with a subsequent release of chemical mediation of inflammation that can recruit and activate leukocytes. Activated leukocytes can produce high levels of ROS in semen and (iii) toxic metabolites of cigarette smoke may impair spermatogenesis, resulting in the production of abnormal spermatozoa which is an important source of ROS and oxidative stress (60, 64). Zn therapy improved sperm quality, increased seminal levels of interleukin-4, and decreased tumor necrosis factor-α and -γ interferon. When a dietary Zn-deficiency was fed to the animals, this caused cadmium accumulation in their testicles in similar amounts to that seen in animals that were given cadmium supplements. The investigators of this study defined that, due to the ability of Zn to enhance Th-2 cytokines and down-regulate Th-1 cytokines, Zn may moderate the putative effects of cadmium on spermatogenesis (31, 65).

### 4. Cellular and molecular mechanisms of Zn

Many studies have focused on Zn homeostasis and its biological relevance. So that recent development in cell biology and the existence and activity of free or unstable Zn in cellular responses were investigated by chemistry, exclusively its neurotransmitter activity in synaptic vesicles (66). On the cellular level, 30–40% of Zn is localized in the nucleus, 50% in the cytosol and the remaining part is associated with membranes (67).

#### 4.1. Interactions of Zn and seminal plasma proteins:

In mammalians, Zn ions are bound to seminal plasma proteins and protect the stability of sperm chromatin. This ion takes part in the formation of S-Zn-S type bonds in protamine structure, which additionally stabilizes chromatin (68). Zn is secreted by human prostate gland in two forms: free and associated with high molecular weight protein complexes (69). Zn is highly concentrated in the tail of mature spermatozoa and is closely associated with sulfhydryl groups and disulfide linkages (68). Zn acts as the regulator of disulfide cross-links in the sperm nucleus by forming a precise number of SH-Zn-SH structures (70). Best known Zn binding proteins of human seminal plasma are semenogelins. They take part in coagulum formation, DNA stability regulation, sperm movement inhibition and antibacterial activity possession. These proteins also, hyperpolarize sperm plasma membranes and prevent capacitation (69).

#### 4.2. Process of Zn transport:

**Figure 2.**
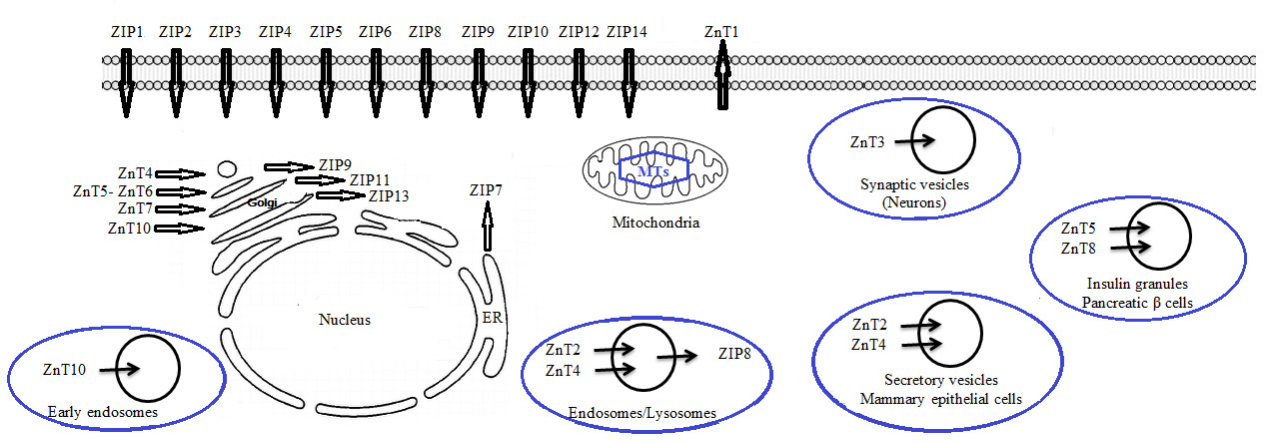
Scheme for zinc dissemination in cells: This scheme is a mixed one obtained from previous studies (21, 67, 74)

Despite the role of Zn for spermatogenesis, testicular function and fertility, there is little understanding of how the testis obtains Zn from circulation, how Zn is transported via the testis in to the maturing gametes during spermatogenesis, or how marginal Zn deficiency influences this process. There are three main mechanisms for cellular Zn transportation. One of them is transport by Zip-family, and export of proteins from the ZnT-family through the plasma membrane. Other one is transmission by Zn-binding proteins such as metallothionein and ultimately third mechanism accomplishment by transporter-mediated decomposition into intracellular organelles, containing endoplasmic reticulum, Golgi, and lysosomes (14). Figure 2 shows two classes of Zn transition proteins motion of Zn across physiological membranes. These proteins work together to protect the proper intracellular Zn concentration. These families of proteins are the SLC30 (ZnT) and SLC39 (Zip). ZnT transfer- or amplifies cellular Zn effluent or its secretion into intracellular organelles. In contrast, Zip transporters make extracellular or intra-organ Zn entrance into the cytoplasm easy (71). Up to now, 10 ZnT and 14 Zip transporters have been known although, only a finite number of studies have inspected human primary tissues from expression patterns of Zn transporters (31). Zip proteins simplify Zn uptake into the cytoplasm, either into the cell from the extracellular environment or into the cytoplasm across an intracellular membrane. Based on our knowledge, expression of only 2 Zn importers, Zip8 and Zip14, has been established in testis (26, 72). Croxford et al. also showed how Zn is transferred from Sertoli cells into the developing gametes during spermatogenesis. Moreover, Croxford et al.’s results combined with data from Song et al. provide compelling evidence that marginal Zn deficiency may be associated with compromised testis function and perhaps infertility in men (26, 73).

#### 4.3. The role of Zn in cell death:

Endogenous zinc plays a remarkable role in cytotoxic events in single cells such as systemic homeostasis and impressive regulatory mechanisms in cellular level and generally hampers the imbibition of cytotoxic doses of exogenous zinc. Zinc via acting on several molecular regulators of programmed cell death, including caspases and proteins from the Bcl and Bax families, affects apoptosis (75, 76).

Table 6 shows that emancipation of intracellular Zn leads to apoptosis. Zn induces mitochondrial apoptogenesis in Zn-accumulating prostate cells. It is now evident that Zn exhibits a direct effect on mitochondria that facilitates the insertion of resident Bax, which is involved in the mitochondrial pore-forming process (77); and is consistent with the release of cytochrome c that also occurs in response to Zn treatment (78). Similarly, Zn treatment induces an increase in cellular Bax levels and Bax/Bcl-2 ratio would bolster the Zn influence on mitochondrial Bax. The increment in cellular Bax provides more Bax for translocation to the mitochondria; and the enhancement in the Bax/Bcl-2 ratio reduces the anti-apoptotic effect of Bcl-2. Zn induction of an increase in cellular Bax and in the Bax/BCL2 ratio has been shown in other cells (79).

**Table 6.** The intracellular and special roles of zinc^*^

**Maintenance of cellular vitality**	**Cell death**
Catalytic, co-catalytic and structural functions	Decrease in Bcl-2/Bax ratio and release of cytochrome-c
Signal transduction	Activation of proapoptotic molecules like p38
Protection from oxidative stress	Activation of potassium channels
Inhibition of caspases	Inhibition of energy metabolism
Increase Bcl-2/Bax ratio	Induction of oxidative stress

* This table is obtained from previous studies (14, 81, 82)

Broadly speaking, regulating the growth and apoptosis of prostate epithelial cells is the role of the zinc in prostate. Therefore, Zn is very essential for maintaining the stability of sperm chromatin and membrane, and also for inhibiting apoptosis for normal sperm morphology (80).

## Conclusion

Adequate Zn content of seminal plasma is needed for men’s health, germination, normal sperm function and fertilization. In contrast, highly toxic content of Zn may have negative effect on sperm quality. Although it certainly cannot be said that seminal Zn deficiency causes infertility, many studies prove that the association of the seminal plasma Zn concentration with physiological and pathogenesis roles of sperm and its quality parameters indicates that Zn deficiency is a menace for sperm dysfunction and male fertility. On the other hand, levels of seminal plasma in Zn may contribute to the effect of cigarette smoking on sperm parameters. Smokers and infertile men with increased ROS levels are susceptible to Zn deficiency in semen, decrease in antioxidant levels and increase in oxidative stress in their seminal fluid. Smokers may not experience decreased fertility but smokers and infertile men with severe Zn deficiency, if they quit smoking or take Zn supplementation, can benefit from it. Therefore, further studies, especially in men with a history of infertility, are required to prove this claim and the assessment of seminal Zn level in IVF center will help in such cases.
